# Small molecule dual-inhibitors of TRPV4 and TRPA1 for attenuation of inflammation and pain

**DOI:** 10.1038/srep26894

**Published:** 2016-06-01

**Authors:** Patrick Kanju, Yong Chen, Whasil Lee, Michele Yeo, Suk Hee Lee, Joelle Romac, Rafiq Shahid, Ping Fan, David M. Gooden, Sidney A. Simon, Ivan Spasojevic, Robert A. Mook, Rodger A. Liddle, Farshid Guilak, Wolfgang B. Liedtke

**Affiliations:** 1 Dept of Neurology, Duke University, Durham NC USA; 2 Dept of Medicine, Duke University, Durham NC USA; 3 Dept of Chemistry, Duke University, Durham NC USA; 4 Dept of Neurobiology, Duke University, Durham NC USA; 5 Dept of Orthopedic Surgery, Washington University in St Louis and Shriners Hospitals for Children, St Louis MO USA.; 6 Dept of Anesthesiology, Duke University, Durham NC USA; 7 Neurology Clinics for Headache, Head-Pain and Trigeminal Sensory Disorders, Duke University, Durham NC USA

## Abstract

TRPV4 ion channels represent osmo-mechano-TRP channels with pleiotropic function and wide-spread expression. One of the critical functions of TRPV4 in this spectrum is its involvement in pain and inflammation. However, few small-molecule inhibitors of TRPV4 are available. Here we developed TRPV4-inhibitory molecules based on modifications of a known TRPV4-selective tool-compound, GSK205. We not only increased TRPV4-inhibitory potency, but surprisingly also generated two compounds that potently co-inhibit TRPA1, known to function as chemical sensor of noxious and irritant signaling. We demonstrate TRPV4 inhibition by these compounds in primary cells with known TRPV4 expression - articular chondrocytes and astrocytes. Importantly, our novel compounds attenuate pain behavior in a trigeminal irritant pain model that is known to rely on TRPV4 and TRPA1. Furthermore, our novel dual-channel blocker inhibited inflammation and pain-associated behavior in a model of acute pancreatitis – known to also rely on TRPV4 and TRPA1. Our results illustrate proof of a novel concept inherent in our prototype compounds of a drug that targets two functionally-related TRP channels, and thus can be used to combat isoforms of pain and inflammation *in-vivo* that involve more than one TRP channel. This approach could provide a novel paradigm for treating other relevant health conditions.

Transient receptor potential Vanilloid 4 (TRPV4) ion channels were initially discovered as osmotically-activated channels^1^,^2^. Discussing the channel’s possible role as mechanosensor, and its expression in sensory neurons in the trigeminal and dorsal root ganglion^1^,^3^,^4^, led to postulation and eventual experimental validation of a possible function in pain sensing and signaling^1^,^3^,^4^,^5^. This medically-relevant role was corroborated over time^6^,^7^,^8^,^9^,^10^,^11^,^12^,^13^,^14^,^15^, as was the mechano-sensory role of TRPV4^11^,^16^,^17^,^18^,^19^,^20^. The pro-nociceptive prostanoid PGE2, activation of PAR-2 signaling, inflammation and nerve injury were found to augment TRPV4-mediated pain signaling in various systems^5^,^6^,^9^,^12^,^21^,^22^, including a novel model of temporo-mandibular joint (TMJ) pain^14^. In a shift of paradigm, TRPV4 was found to function as a relevant sensing molecule in epidermal keratinocytes for UVB overexposure^15^. UVB-exposed keratinocytes, depending on their TRPV4 expression and signaling, were functioning as organismal pain generators, supported by the finding that deletion of *Trpv4* exclusively in these cells sufficed to greatly attenuate the organismal pain response. TRPV4 was also found to play a role in visceral pain, e.g. of the colon and pancreas^7^,^8^,^18^,^23^,^24^,^25^, the latter two conditions also co-involving TRPA1^8^,^24^,^26^,^27^,^28^. The co-involvement of TRPV4 and TRPA1 was also noted in our TMJ model^14^, as well as in formalin-mediated irritant pain of the trigeminal territory, which serves as a generic model of cranio-facial pain^13^.

Importantly, blocking TRPV4 with selective inhibitors shows similar results as those obtained with genetic knockouts^13^,^14^,^25^,^29^,^30^,^31^,^32^,^33^,^34^, particular in models of TMJ pain or formalin-induced trigeminal formalin pain^13^,^14^. These findings suggest that TRPV4 could serve as a critical pain target, thus incentivizing the development of more potent and selective small-molecule inhibitors as new clinically-relevant therapeutic drugs. This direction has advantageous features because genetic approaches are currently limited to experimental conditions and TRPV4 inhibitors are not yet clinically available

The goal of this study was to develop TRPV4 inhibitors with increased potency over a previously used tool compound, GSK205^32^,^33^,^34^. Our results indicate that we have successfully developed compounds with significantly increased TRPV4-inhibitory potency as compared to the tool compound. Interestingly, our approach led to the development of two novel inhibitor molecules that simultaneously target TRPV4 and TRPA1, a potentially advantageous property that we successfully applied in two exemplary *in-vivo* preclinical models of pain, irritation and inflammation.

## Results

### Chemical synthesis of GSK205 derivatives and assessment of their TRPV4-inhibitory potency in cell-based assays

**Figure 1:**
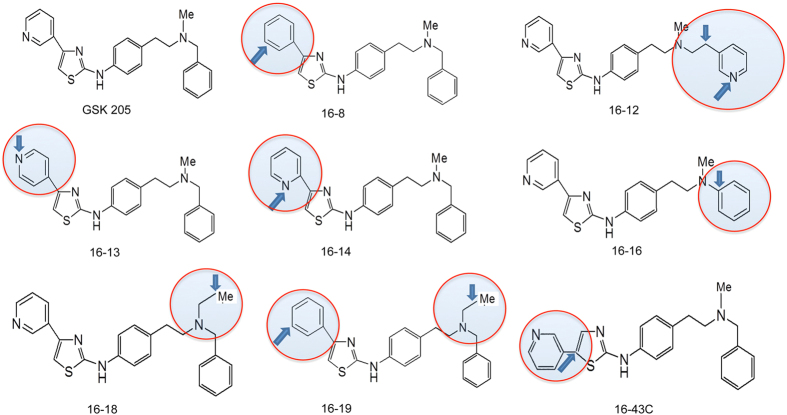
Modifications of tool compound GSK205 for improved targeting of TRPV4. The synthesized compounds differed in the highlighted part of the molecule, changed residue indicated with arrow. Compound 16-19 compound was synthesized to incorporate two modifications from two compounds, 16-8 and 16-18, found most potent in anti-TRPV4 screening assays (see Fig. 2).

**Figure 2:**
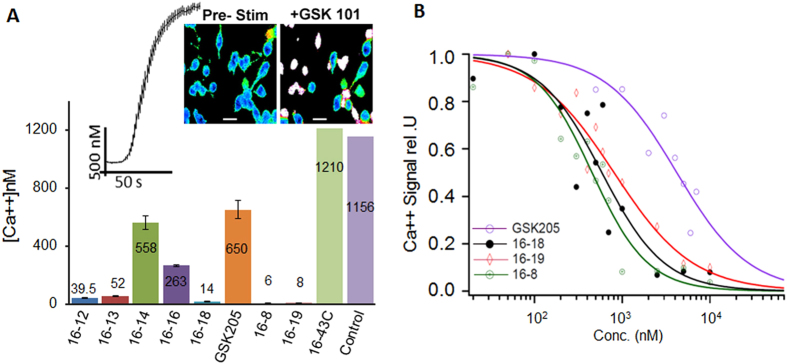
Assessment of 16-… compounds in N2a cells with directed expression of TRPV4. (**A**) Ca^++^ imaging screening of all compounds in N2A cells with directed expression of TRPV4 (rat). The cells were stimulated with TRPV4-selective activator compound, GSK101 (5 nM) in the presence of 5 μM of the respective inhibitor. The number on each bar corresponds to average peak ∆Ca^++^ concentrations in ≈100 cells. Inset: micrographs of pseudo-colored cells before and after activation with 5nM GSK101, in addition note the corresponding time course of the averaged Ca^++^ signal (fura-2 Ca^++^ imaging). Except for compound 16-43C, the difference to vehicle control reach the level of statistical significance p < 0.01 (one-way ANOVA). **(B)** Dose-response of the most potent, “winner” compounds in TRPV4-expressing N2a cells. The IC_50_ were; 0.45 ± 0.05 μM (16-8), 0.59 ± 0.12 μM (16-18), 0.81 ± 0.1 μM (16-19), 4.19 ± 0.71 μM (GSK205). Plot generated from averaged peak ∆Ca^++^ concentration of ≥75 cells per data-point.

We modified compound GSK205 by generating 7 primary modifications, as shown in Fig. 1. One additional compound (16-19) that had the combined respective modifications of the two most potent compounds, as defined in primary screens, was also synthesized. We assessed TRPV4-inhibitory potency of these synthetic compounds in a Ca^++^ imaging assay in neuronal 2a (N2a) permanent tissue culture cells with directed expression of mammalian (rat) TRPV4. TRPV4 channels were stimulated with a selective activator compound, GSK1016790A (GSK101), used at 5 nM. For first round assessment, all TRPV4-inhibitory compounds were used at 5 μM (Fig. 2A). Compound 16-43C did not inhibit Ca^++^ influx, and its effect was similar to vehicle control. All other compounds inhibited TRPV4-mediated Ca^++^ influx, with compounds 16-8 and 16-18 emerging as the two most potent. Compound 16-19 which incorporated the modifications of both 16-8 and 16-18, was also effective in inhibiting TRPV4-mediated currents. However, we did not find a significant difference between compound 16-19 and 16-8, both of which virtually eliminated Ca^++^ influx.

**Figure 3:**
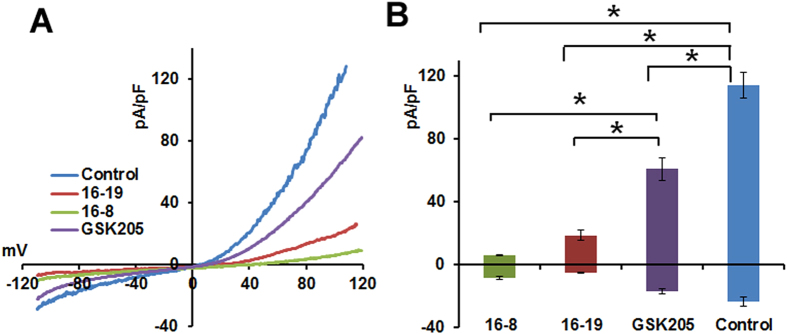
TRPV4 channel inhibition by compounds 16-8 and 16-19 – patch-clamp e-phys. **(A)** Current-voltage relationship of TRPV4-mediated currents after activation with 5 nM GSK101. Recordings were performed in TRPV4-GFP+ N2a cells. The representative traces represent an average of ≈12 sweeps. In all experiments, cells were pre-incubated with the respective compound (5 μM) for 5 minutes. (**B)** Average current densities at −100mV/+100 mV were significantly diminished by inhibitors (*P < 0.05; one-way ANOVA; n ≥ 5 cells/group).

We then conducted more detailed dose-response assessments for compounds 16-8, 16-18 and 16-19, which yielded an IC_50_ of 0.45, 0.81 and 0.59 μM, respectively, vs. an IC_50_ of 4.19 μM for parental compound GSK205. These findings represent an increased potency of the GSK205-derivative compounds by approximately 10-fold for 16-8, 8-fold for 16-19 and 5-fold for 16-18. Surprisingly, compound 16-19 was not significantly more potent than 16-8, whereas 16-8 was more potent than 16-18. Based on these results, we tested 16-8 and 16-19 vs GSK205 (as a control) in patch-clamp studies (Fig. 3). Our results indicate significantly increased potency of compounds 16-8 and 16-19 as compared to the parental molecule, GSK205 (all applied at 5 μM) in attenuating TRPV4-mediated currents.

**Figure 4:**
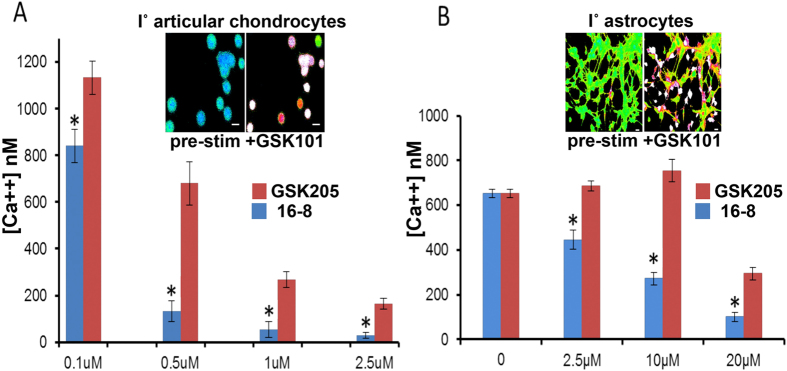
Compound 16-8 inhibits TRPV4 in I˚ cells more potently than GSK205. **(A)** I˚ articular chondrocytes (pig); dose-response comparison between the most potent compound, 16-8, and GSK205 in response to stimulation with 5 nM GSK101. Inset: Chondrocytes responding to activation with GSK101, fura-2 Ca^++^ imaging; right-hand image taken at 5 sec after GSK101 application. 16-8 was significantly more potent than GSK205 (mean ± SEM, n = 6 independent expts, n ≥ 25 cells/expt; *p < 0.05, t-test). Ordinate shows average peak ∆Ca^++^ concentrations. **(B)** I˚ astrocytes (rat); dose-response comparison between 16-8 and GSK205 in response to 5 nM GSK101. Inset: Astrocytes responding to activation with GSK101; right-hand image taken at 5 sec after GSK101 application (mean ± SEM, n = 5 independent expts, n ≥ 200 cells/expt; *p < 0.05, t-test). Ordinate shows average peak ∆Ca^++^ concentrations.

We next decided to assess potency of the most potent compound 16-8 vs. parental compound GKS205 in two types of primary cells that are known to express TRPV4 and in which TRPV4 function has been demonstrated in a relevant biological context. We examined articular chondrocytes, which have prominent TRPV4 expression, where TRPV4 serves as the mechanotransducer of physiologic mechanical loads to regulate the cells’ anabolic response, and thus tissue homeostasis, in cartilage^19^. In addition, we studied brain astrocytes, where TRPV4 expression and relevant function has been previously demonstrated in regulating astrocyte cellular edema, in the coupling of neuronal activity to cerebral blood flow, and in mediating CNS traumatic injury^35^,^36^,^37^. Fulfilling our main objective, in both cell types we observed significantly increased potency of compound 16-8 as compared to the parental molecule, GSK205 (Fig. 4). Evaluation of the inhibitory potency of GSK205 derivatives in these primary cells, which express functional and biologically-relevant TRPV4 without directed over-expression of the channel (Fig. 4), directly corroborate the findings of more basic studies using heterologously TRPV4-overexpressing immortalized cell lines (Fig. 2), strongly supporting our conclusions on the increased potency of the newly derived compounds. Taken together, we identified compound 16-8 as a TRPV4-inhibitory compound with sub-micromolar potency in heterologous systems, with approximately a 10-fold increase in potency as compared to its parental molecule, GSK205. Moreover, 16-8 proved more effective in TRPV4-expressing primary skeletal and CNS-derived cells. However, the rational modification to compound 16-19, intended to further enhance potency, did not yield the intended effect.

Before testing these compounds in relevant *in-vivo* animal models, we next tested their cellular toxicity as well as the specificity of these compounds against other selected TRP ion channels.

### Novel TRPV4 inhibitors are selective, with benign toxicity profile, yet display potent inhibition of TRPA1

**Figure 5:**
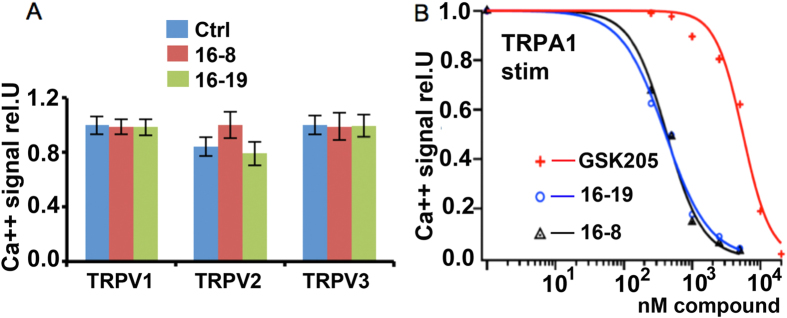
Compounds 16-8 and 16-19 also potently inhibit TRPA1, not TRPV1-3. **(A)** Specificity vs TRPV1-3. Both 16-8 and 16-19 (5 μM each) compounds did not inhibit TRPV1, −2 or −3 channels (all mouse isoforms), directed over-expression in N2a cells and subsequent Ca^++^ imaging. Mean±SEM is shown, ≥100 cells per condition. **(B)** Dose-dependent inhibition of TRPA1 (mouse, directed expression in N2a cells) by GSK 205, 16-8 and 16-19, activation with 100 μM mustard oil, resulting in IC_50_ of 5.56 ± 0.4 μM (GSK205), 0.41 ± 0.37 μM (16-19), 0.43 ± 0.3 μM (16-8). Plot generated from averaged peak ∆Ca^++^ concentration of ≥75 cells per data-point.

In heterologously transfected permanent N2a cells, we did not observe inhibitory potency of compounds 16-8 or 16-19 toward TRPV1, TRPV2 and TRPV3 (Fig. 5A). However, we made the unexpected discovery of sub-micromolar inhibitory potency vs TRPA1 for compounds 16-8 and 16-19, micromolar potency for GSK205, and, remarkably, no significant activity for compound 16-18 (Fig. 5B). We recorded IC_50_ of 0.41, 0.43, 5.56 μM and >25 μM for compounds 16-8, 16-19, GSK205 and 16-18, respectively.

**Figure 6:**
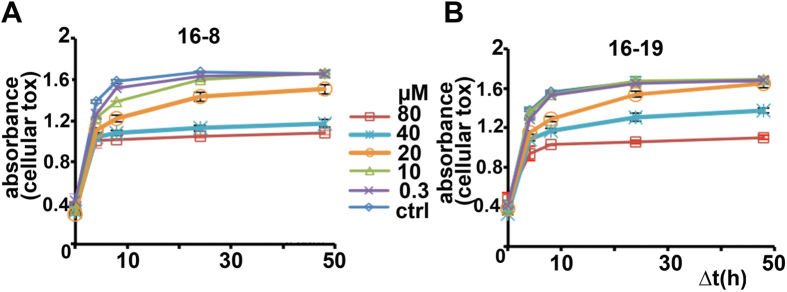
Cellular toxicity studies of compounds 16-8 and 16-19. N2a cells were subjected to increasing concentrations of compounds 16-8 and 16-19, resulting cell viability was analyzed for the next 48 h. **(A)** Time course of cell viability in the presence of various concentrations of 16-8. Note clear reduction at 40 and 80 μM. **(B)** As in (**A**), for compound 16-19, with similar outcome. Representative result of 2 independent experiments.

In terms of cellular toxicity, we found first evidence of toxicity using a sensitive cell viability assay over a time-course of 2 days, at 20 μM, and more pronounced effects at 40 μM (Fig. 6).

Taken together, our results indicate that compounds 16-8 and 16-19 also inhibit TRPA1 at sub-micromolar potency, and their cellular toxicity vs their inhibitory potency against TRPV4/TRPA1 ranges at a factor of 50–100. Assessment of specificity of 16-8, 16-18 and 16-19 against a wider spectrum of receptors and ion channels will be the subject of dedicated future studies directed toward translation of these compounds to the clinic.

We next evaluated these compounds to an *in-vivo* model of irritant pain known to rely on both, TRPV4 and TRPA1.

### TRPV4/TRPA1 dual-inhibitors are effective in containing trigeminal irritant pain

We have previously described TRPV4 as a cellular receptor for formalin^13^, and demonstrated its involvement in formalin irritant-evoked pain behavior, with a focus on trigeminally-mediated irritant pain behavior. In this recent study, we also demonstrate the co-contribution of TRPV4 together with TRPA1 in trigeminal formalin-evoked pain. We also showed the effective attenuation of trigeminal formalin-evoked pain behavior using GSK205 in a dose-dependent manner. Moreover, we found irritant pain behavior in response to selective activation of TRPV4 in the trigeminal territory, which was blocked by GSK205, and the absence of such an effect in *Trpv4*^−/−^ pan-null mice.

**Figure 7:**
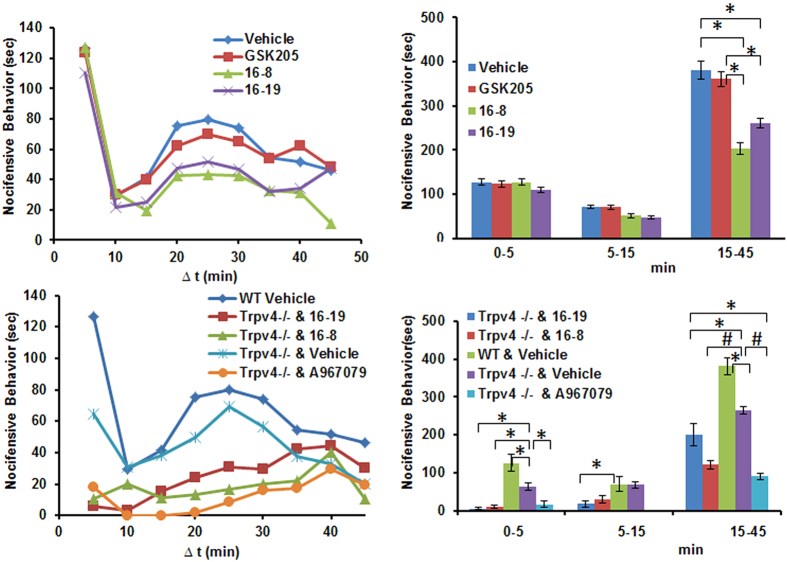
16-8 and 16-19 effectively attenuate formalin-evoked trigeminal irritant pain. **(A)** Time-course of nocifensive behavior in WT mice following whisker-pad injection of 4% formalin. The mice were pre-injected (i.p., 10 mg/kg; 15 min before formalin) with GSK205, 16-8 or 16-19. Note effective reduction of nocifensive behavior in the late “neural” phase by compounds 16-8, 16-19, not by GSK205. **(B)** Cumulative response binned into 3 phases: acute phase (0–5 min), interphase (5–15 min), and late “neural” phase (15–45 min). Note significant reduction of nocifensive behavior in the late phase by 16-8, 16-19, not GSK205 (*P < 0.01 vs vehicle and GSK205, one-way ANOVA). **(C)** As in (**A**), but also including *Trpv4*^−/−^ mice. Compounds were applied i.p. 15 min before formalin challenge, at 10 mg/kg except established TRPA1 blocker, A967079 (25 mg/kg). Previously-established attenuated nocifensive behavior in early and late phase in *Trpv4*^−/−^ mice was recapped, which was reduced further by TRPA1 blocker, A967079. **(D)** As in (**B**), plus inclusion of *Trpv4*^−/−^ mice. Robust effects of TRPA1-blocker, A967079, were mimicked equi-potently by 16-8 and 16-19 for early phase, and by 16-8 for late phase, partially by 16-19 for late phase. (**A**,**C**) show averaged behavioral metrics per time-point, bars in (**B,D**) represent mean ± SEM; for (D) ^*^P < 0.05; ^#^P < 0.005, one-way ANOVA; for all panels n = 5–8 mice/group.

With this pertinent background as a rationale, we applied TRPV4/TRPA1 dual-inhibitory compounds 16-8 and 16-19 systemically, using GSK205 as control, at 10 mg/kg dosage. We prioritized the dual-inhibitors over testing of compound 16-18 (TRPV4-only inhibitor) because (i) the trigeminal formalin pain model relies on both TRPV4 and TRPA1, (ii) we intended to develop TRPV4/TRPA1 dual-inhibitory molecules toward translational use in the first place. None of the compounds were effective at significantly diminishing pain behavior in the early phase after formalin whisker-pad injection, which represents an acute chemical tissue injury pain. In the delayed phase, which is understood as neurally-mediated pain indicative of early maladaptive neural plasticity, there was a significant attenuation of formalin-evoked pain behavior in response to compound 16-8 and 16-19, with compound 16-8 diminishing pain behavior at a remarkable >50%^13^, and compound 16-19 also showing a robust effect (Fig. 7A,B). Of note, at 10 mg/kg systemic application, there was no significant effect of GSK205, which was effective previously in a dose-dependent manner when applied by intradermal injection^13^. Thus, compounds 16-8 and 16-19, upon systemic application, effectively attenuate the late, neurally-mediated phase of trigeminal formalin pain, and these compounds are more potent *in-vivo* than their parental compound, GSK205.

In view of these *in-vivo* findings, taken together with the results from heterologous cellular expression systems that indicate an additional TRPA1-inhibitory effect of compounds 16-8 and 16-19, we decided to assess effectiveness of these compounds in a setting of genetically-encoded absence of *Trpv4* (*Trpv4*^−/−^ mouse), in order to better define their TRPA1-inhibitory potency *in-vivo*. We observed significant residual irritant-pain behavior in all phases of the formalin model in *Trpv4*^−/−^ mice, consistent with our previous report^13^ (Fig. 7C,D). Immediate-phase pain behavior was virtually eliminated with compounds 16-8 and 16-19, both applied again at 10 mg/kg body-weight. Late-phase pain behavior was strikingly reduced when applying compound 16-8, and still significantly reduced vs vehicle-treated *Trpv4*^−/−^ when applying compound 16-19, although not as potently as 16-8. A reference TRPA1-inhibitory compound, A967079, was used at 25 mg/kg body weight as a positive control to inhibit TRPA1, based on a previous report^38^. Reduction of pain behavior in *Trpv4*^−/−^ mice was striking, more than 50% in the late neural phase. We noted equal potency of compound 16-8 at 10 mg/kg body weight vs. reference TRPA1-inhibitory compound A967079 at 25 mg/kg body weight, both reducing formalin-evoked trigeminal pain behavior to similarly robust degree (Fig. 7C,D). We conclude that compound 16-8 also functions as a potent TRPA1-inhibitor in an *in-vivo* irritant pain model specifically designed to assess the contribution of TRPA1 to trigeminal irritant pain, and at least as potent as an established reference TRPA1-antagonistic compound.

### Potent TRPV4/TRPA1 dual-inhibitor, 16-8, is effective at controlling inflammation and pain in acute pancreatitis

These findings define compound 16-8 as a potent TRPV4/TRPA1 dual-inhibitor molecule, based on cell-based and live-animal results. We therefore decided to test it in a more specific preclinical pain model that relies on both, TRPV4 and TRPA1, in order to establish proof-of-principle that a dual-inhibitor can effectively treat pain and inflammation in pancreatitis. We used a pancreatitis model because it provides high translation potential due to unmet clinical need for new effective treatments in this condition^39^, as well as the known role of both TRPV4 and TRPA1 in pancreatitis pain and inflammation^8^,^25^,^40^.

**Figure 8:**
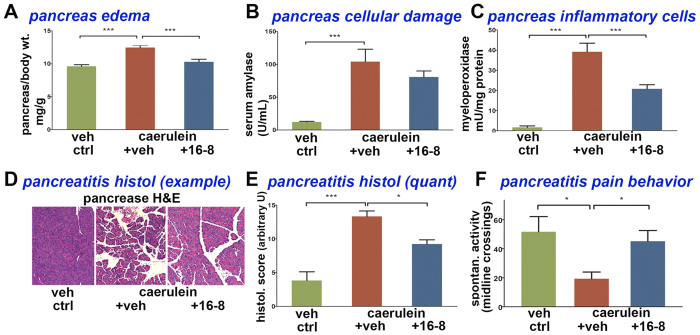
Compound 16-8 attenuates acute pancreatitis and improves pain behavior. **(A)** Caerulein-evoked acute pancreatitis causes pancreatic edema, which is eliminated by compound 16-8 (10 mg/kg, applied at 30 min before first exposure to caerulein). **(B)** Caerulein-evoked acute pancreatitis strongly elevates cellular toxicity marker amylase in serum. Amylase is reduced, but not significantly, in 16-8 treated animals. **(C)** caerulein-evoked acute pancreatitis causes elevated myelo-peroxidase (MPO) activity in serum, a marker for infiltration of inflammatory cells into the pancreas. MPO activity is significantly reduced in 16-8 treated mice. **(D)** caerulein-evoked acute pancreatitis can be readily demonstrated histologically, exemplified in the micrograph panels shown. Note increased pancreas inflammation in the middle-panel vs non-inflamed pancreas in vehicle-control challenged mice, and its attenuation by treatment with compound 16-8. **(E)** Bar diagram shows quantitation of inflammatory histologic parameters as shown in (**D**). Note significant increase of inflammation-index in caerulein acute pancreatitis mice, and its significant reduction upon treatment with compound 16-8. **(F)** Caerulein-evoked acute pancreatitis causes pain behavior, significantly reduced by compound 16-8. Note greatly reduced activity over the 6 h test period in caerulein-induced acute pancreatitis. This nocifensive behavior is greatly improved in response to systemic application of compound 16-8. Results are expressed as mean ± SEM; n = 6 mice/group; *P < 0.05 (one-way ANOVA).

Pancreatitis was induced with caerulein, a well-characterized model for acute pancreatitis^41^. Animals were treated with 10 mg/kg bw 16-8 by intraperitoneal injection, 30 min before induction of inflammation. We found significant attenuation of inflammatory parameters, namely edema, which was virtually eliminated in 16-8 treated animals. Furthermore, serum amylase, a marker of inflammatory injury of the pancreas, was significantly reduced by 16-8 treatment, as was myelo-peroxidase content of the pancreas, as a marker of inflammatory cell infiltration of the pancreas. The histopathological score for pancreas inflammation was also significantly reduced (Fig. 8A–E). Of note, pain behavior, similar to the effect of 16-8 on pancreas edema, was virtually eliminated upon treatment with compound 16-8. Thus, compound 16-8 was found to be highly effective in attenuating pain and inflammation of acute chemically-evoked pancreatitis.

### Benign preliminary toxicity and pharmacokinetics of novel TPRV4/TRPA1 inhibitors

Promising properties of potent 16-… compounds prompted us to attempt to define their initial preliminary features in terms of *in-vivo* toxicity and pharmacokinetics, which will be followed by more extensive and in-depth investigations in future studies. For this, we chose compound 16-8 as the all-around most potent dual TRPV4/TRPA1 inhibitor, and also compound 16-19 with its potentially increased lipophilicity (Suppl Table 1). We measured compound concentration in several organs and plasma, and detected micromolar/submicromolar concentrations in liver and kidney, less than 100 nM concentrations in heart, brain, brainstem, trigeminal ganglion and skin. Of note, compounds were virtually undetectable in plasma (Suppl Fig. 1A). We detected higher concentrations of 16-19 in non-nervous tissue, especially liver and kidney, a pattern perhaps related to compound 16-19’s increased lipophilicity. Based on this finding, we next examined 6 and 24 h time-points and detected 10-20 fold higher concentrations of 16-19 at the 6 h time-point, compared to the 1 h time-point, indicative of compound sequestration into solid organs (Suppl Fig. 1B–D). Values at the 24 h time-point were lower than at the 6 h but higher than at the 1 h time-point, indicating ongoing protracted compound clearance. We next confirmed that low/non-detectable concentrations in plasma were not caused by compound denaturation/degradation in plasma, as indicated in Suppl Fig. 1E, which shows no loss of detectable compound after 4 h incubation in plasma at 37 °C, conducted using compound 16-19.

We next performed basic preliminary *in-vivo* toxicity studies for compounds 16-8 and 16-19, both at 10 mg/kg bw, which was the effective concentration in both *in-vivo* pain models. We did not detect first evidence of cardiac, hepatic and renal toxicity, when comparing compounds 16-8 and 16-19 with vehicle (Suppl Fig. 2). For cardiac assessment, we did not detect differences and changes in heart rate over 1 h, conducted by EKG at the 6 h time-point. Serum creatinin as marker of renal function and alanine-amino-transferase (ALT) as marker of hepato-cellular integrity were not significantly elevated in animals treated with compounds 16-8 or 16-19. Thus, initial evidence for potent 16-… compounds highlights their acceptable preliminary pharmacokinetics properties as well as lack of gross systemic toxicity. Future studies will be necessary for more detailed assessment of the pharmacokinetics and toxicity of these compounds.

## Discussion

Here we describe novel compounds that simultaneously inhibit both TRPV4 and TRPA1 ion channels. Both targets were inhibited by the novel “dual-inhibitors” at sub-micromolar potency in heterologous cellular channel activation assays. Furthermore, these compounds showed potent activity against TRPV4 in primary cells with native TRPV4 expression, and were more potent than their related parent-compound. The most potent compound identified here, compound 16-8, also showed a favorable activity profile in two pain-inflammation models, one of them a general irritant-pain model in the trigeminal system, the trigeminal formalin model, the other a visceral pain and inflammation assay with specificity for the pancreas, the caerulein-induced acute pancreatitis model. Of note, both *in-vivo* pain models have been shown previously to rely on co-contribution of TRPV4 and TRPA1. In this regard, several other relevant medical conditions, discussed in more detail below, also rely on TRPV4/TRPA1 and represent important unmet clinical needs that need to be addressed by translational-medical approaches. Therefore a potent “dual-inhibitor” for a specific combination of TRP ion channels, such as TRPV4 and TRPA1, could be highly beneficial in these indications.

More specifically, primary chondrocytes represent a cellular model for joint disease with involvement of TRPV4 in cartilage maintenance as well as arthritis/osteoarthritis. Recent evidence also suggests a potential role for TRPA1 in mediating joint pain in osteoarthritis^42^. In addition, TRPV4-expressing astrocytes are involved in many neurologic and psychiatric diseases such as pain, epilepsy, multiple sclerosis and other CNS autoimmune conditions, stroke, traumatic brain/spinal cord injury, brain edema, CNS infections, and more^35^,^36^,^37^. TRPA1 expression in astrocytes has also been reported^43^. Therefore, novel dual TRPV4/TRPA1 inhibitors might be suitable compounds for treatment of a number of diseases, from a spectrum of disorders affecting the nervous system as well as degenerative or inflammatory musculoskeletal conditions. For both types of disorders, we view compartmentalized application of compounds, i.e. intra-thecal or intra-articular delivery, as feasible future routes of delivery, in order to affect target cells more directly without affecting - TRPV4 and TRPA1 systemically.

In terms of *in-vivo* pain models, the trigeminal formalin model is rather a general model, not a direct pre-clinical translational-medical model. However, formalin models have a very robust track record in the identification of efficacious new compounds against pain^44^. Our findings with compound 16-8 in the trigeminal formalin model can be interpreted along these lines, especially for trigeminal pain including headaches^45^,^46^. In the context of these most prevalent neurological diseases, involvement of TRPV4 and TRPA1 has been reported previously in preclinical models, underscoring the case for their involvement with some compelling preclinical insights for both channels^47^,^48^,^49^,^50^.

Pancreatitis in mice represents a more specific preclinical visceral inflammatory pain model, helpful to elucidate pathophysiology of this specific pain in order to better address a significant unmet medical need. In the current study pain and edema were strongly reduced by compound 16-8, the compound identified as showing the most advantageous features when tested in the trigeminal formalin pain model vs another high-potency dual-inhibitor, 16-19, and vs parent molecule, GSK205. This is in keeping with previous studies which demonstrated that both TRPA1 and TRPV4 contributed to pancreatic inflammation and pancreatic pain^25^. More precisely TRPA1 was shown to contribute to both pain and inflammation while TRPV4 contributed more selectively toward pain^8^. In our current study, there was significant attenuation of inflammatory parameters, but the reduction was not as robust as for pain and edema. This finding begets two important issues, namely (i) this could be a feature of the 2 targeted TRP channels, that they are more significant for pain than for inflammation (as concluded from experimental evidence in^14^), and (ii) edema of the pancreas, which is readily measurable by imaging techniques in patients, could possibly serve as a bio-marker for pancreatic pain. Beyond its role as biomarker, pancreas edema could sustain pancreas pain. At the pathophysiological level, edema will encompass edematous distension of the inflamed organ causing mechanical pain. This pain, amplified by inflammation of the painful organ, will in turn cause neurogenic inflammation, which will give rise to increased level of edema. This could evolve into a detrimental feed-forward mechanism. The specific effect of compound 16-8 on pancreatitis pain could be due to the compound interfering potently and efficiently with such a feed-forward mechanism as in (ii), by potently inhibiting both channels, as laid out in (i).

Beyond the two pain-inflammation conditions tested, TRPV4/TRPA1 co-involvement appears to play a role in several health-relevant conditions, such as colitis, itch, injury to airway and lungs via the inhalatory route and chronic cough^28^,^34^,^51^,^52^,^53^,^54^,^55^,^56^,^57^,^58^. In addition, interesting recent findings point toward a prominent role for TRPV4 in conditions as diverse as fibrotic disorders, UVB skin injury, and premature birth^15^,^59^,^60^. In these conditions, possibly via TRPA1-expressing innervating sensory neurons, a co-contribution by TRPA1 could be an important element. For such cases, 16-… compounds represent attractive candidates for effective treatment, to address the significant underlying unmet clinical need. Except pancreatitis, compound access to relevant target cells could also be readily accomplished by topical, non-systemic delivery via transdermal, transmucosal, inhalatory, intra-articular or intra-thecal formulations.

Our study presented here was strongly geared toward a translational medical agenda, meaning demonstration of effect of TRPV4/TRPA1 dual inhibitors, combined with a first-pass at pharmaco-tox assessment were our priority, rather than in-depth mechanistic studies. In addition, our goal was to demonstrate that modified 16-… compounds were more potent than the parent compound, GSK205. For future studies, in addition to continuation of a translational-medical agenda based on 16-… compounds, e.g. in-depth assessment of 16-… compounds at human isoforms of TRPV4 and TRPA1, effect of 16-… compounds in TRPV4/TRPA1-expressing primary human cells, our current results raise the following important questions/issues, namely a mandate to conduct mechanistic studies that address how TRPV4/TRPA1 dual inhibitors act on their respective target channels, and whether there is perhaps a shared mechanism between TRPV4 and TRPA1 of channel inhibition by potent 16-… compounds. In this context, it will be rewarding to zero in on a potential mechanism as to why compounds 16-8, 16-19 and to minor degree GSK205 are active against TRPA1 whereas compound 16-18 interestingly is not. Furthermore, the question why compound 16-19 fails to show increased potency over 16-8 will be an interesting one to address in future studies.

## Materials and Methods

### Compound synthesis

Compound synthesis is explicitly described in Supplementary Information (Suppl Fig. 3 and Suppl Tables 2–5).

### Animals

8–12 week old mice were used throughout the experiments. *Trpv4*^−/−^ mice^3^ have been outcrossed to WT (C57BL/6J) background and genotyped by PCR^3^,^15^. Animals were housed in climate-controlled rooms on a 12/12 h light/dark cycle with water and standardized rodent diet that was available ad libitum. All experiments were conducted in compliance and accordance with the guidelines of the NIH and the Institutional Animals’ Care and Use Committee (IACUC) of Duke University, and under a valid IACUC protocol of the Duke University IACUC. All animal methods described in this publication were approved by the Duke University IACUC.

### Trigeminal formalin irritant behavior – mouse model

Implementation of this model was conducted as described previously^13^.

Video-taped nocifensive behavior was assessed by investigators blinded to genotype and treatment.

### Acute pancreatitis mouse model

C57BL/6J male mice 8–10 weeks of age were subjected to acute pancreatitis by intraperitoneal injections of supramaximal doses of caerulein (50 μg/kg) every hour for a total of 6 h, as previously described in^61^. Control animals received 25% DMSO-saline solution by intraperitoneal injection every hour for 6 h. Compound 16-8 was dissolved in this vehicle (10 mg/kg) and injected i.p. 30 min prior to the first injection of caerulein. Animals were sacrificed 1 h after the last injection. Blood was collected and pancreatic tissue was promptly isolated, weighed for determination of pancreas wet weight/body weight ratio. Samples of tissues were fixed overnight in 10% neutral-buffered formalin, paraffin embedded and H&E-stained, or pancreatic tissue was quickly frozen and assessed for myeloperoxidase (MPO) activity. Serum amylase, MPO and histologic evaluation were conducted as described previously^61^.

Assessment of nocifensive behavior^8^: Mice were housed in individual cages and video-recorded during the entire experiment. Two mice at a time were observed. Linear movement was measured as one event when mice passed through the median plane of the cage. Analysis began immediately after the first caerulein/vehicle injection and continued until the end of the experiment. Results were expressed as the sum of the movement events spanning the 6 h time-period following the first injection.

### Cell cultures

N2a cells were used for directed expression of TRP ion channels as described previously^13^. TRPV4-eGFP from rat was used, previously found to respond to stimulation with GSK101 and hypotonicity in similar manner as native, non-fused TRPV4. All other channels were native channels from mouse, eGFP was co-transfected. Stimulation of over-expressed TRPV4 was conducted with GSK101 (5 nM), TRPV1 with capsaicin (10 μM), TRPV2 with hypotonicity (270 mosmol/L), TRPV3 with camphor (100 μM) and TRPA1 with mustard oil (100 μM). eGFP control-transfected N2a cells did not respond to these stimuli. Ca^++^ imaging was performed as described previously^13^,^14^,^34^.

To visualize dose-response relationships, Hill plots were conducted using the Igor Pro software program, which derived the plots based on the following equation:


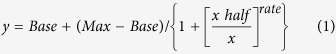


Primary porcine chondrocytes derived from femoral condyles of skeletally mature pigs were cultured and subjected to Ca^++^ imaging as described previously^19^,^32^,^62^,^63^.

Astrocyte cultures were conducted following established protocols^64^,^65^,^66^. Astrocytes were prepared from Sprague Dawley rat embryos (E18). Briefly, the isolated cortices were minced, and then incubated with trypsin and DNase. Dissociated cells were suspended in Dulbeccco’s Modified Eagle’s Medium (DMEM) supplemented with 10% fetal calf serum and penicillin/streptomycin (100 U/ml and 100 μg/ml, respectively). Thereafter, cell suspensions were plated in 75 cm^2^ tissue culture flasks (10 × 10^6^ cells/flask) which were pre-coated with poly-L-lysine (10 μg/ml). The cells were maintained in a 10%CO_2_ incubator at 37 °C. After 10–12 days, the media was removed and adherent cells were trypsinized (0.25%) and plated out onto coverslips for subsequent Ca^++^ imaging^34^,^67^. >95% of the cells were found to express astrocyte marker, glial fibrillary acidic protein (GFAP)^68^.

### Cell viability in culture

N2a cells were cultured in 96 well plates for 24–48 h. Cell viability studies relied on metabolic capability monitored with the indicator dye resazurin. Its reduction to resorufin (indicated by color change dark blue to pink) was monitored over time. Changes in absorbance at λ = 570 nm were recorded using a microplate reader (Molecular Devices). Metabolically active and viable cells shared the ability to reduce resazurin to resorufin whereas dead cells did not. Eight replicate cultures per experimental point were studied.

### Assessment of hepatic, renal and cardiac function in mice treated with 16-… compounds

Mice were treated i.p. with compounds 16-8 and 16-19 (10 mg/kg). Hepatic and renal integrity were analyzed by alanine amino-transferase- and creatinine assays (Sigma), both relying on measurement of absorbance at λ = 570 nm in 96-well micro-titerplates. 8 technical replicates per animal were performed.

For heart rate assessment in mice treated with 16-8 and 16-19, animals were fitted with two electrodes, one to the ear, via clip, one to the rib-cage, using firm adhesive. Heart rate was monitored and analyzed using axoscope and clampfit 9.2 software (Molecular Devices)

### Liquid Chromatography – Tandem Mass Spectrometry (LC-MS/MS)

Mice were treated with 10 mg/kg i.p. of the respective inhibitor. Post-euthanasia harvested tissue was frozen in liquid nitrogen and stored at −80 °C for further analysis.

Frozen tissue samples were partially thawed and cut into 1 mm slices, 5–15 mg tissue, 2-fold excess water (mass/vol.), 6-fold excess acetonitrile (16-… compounds) or methanol (GSK205) containing appropriate amount of internal standard, and 2.5 mm zirconia/silica bead (Biospec Products Inc.) were added to 500- μL polypropylene (PP) conical tube, homogenized in a Fast-Prep apparatus (Thermo-Savant) at speed “4” for 20 sec at room temp, and centrifuged at 13,600 g for 5 min at room temp. Depending on the expected concentration range of the measured compound, the supernatant was diluted 1/4–1/20 (in Mobile phase A, see below) and placed in autosampler for LC-MS/MS analysis.

The LC-MS/MS assay for 16-… compounds and GSK205 was developed on an Agilent 1200 series LC system interfaced with Applied Biosystems API 5500QTrap, a hybrid triple quadruple-linear trap MS/MS spectrometer. Analyst (version 1.6.1) software was used for mass parameters tuning, data acquisition, and quantification. LC column: 3 × 4 mm RP C18 (Phenomenex, AJ0-4287) was operated at 35 °C. Mobile phase A: 0.1% formic acid, 2% acetonitrile, in LC/MS-grade water; mobile phase B: acetonitrile; flow rate: 1 mL/min, 1:1 MS/MS:waste split. Run time was 4 min. Diverter valve was used to send flow to MS/MS only between 1.2 and 2.5 min. The elution gradient was: 0–0.5 min, 1%B; 0.5–1.2 min, 1–95%B; 1.2–1.5 min, 95%B; 1.5–1.6 min, 95–1%B. Autosampler was operated at 4 °C; injection volume was kept at 10–50 μL. Electrospray ionization (ESI) source parameters were: positive ionization mode, curtain gas flow = 30, ionization potential = 5500 V, temperature = 500 °C, nebulizing gas 1 flow = 30, nebulizing gas 2 = 30, declustering potential = 20 V. 16-… compounds and GSK205 were individually infused as 100 nM solutions in 50%A/50%B at 10 μL/min flow rate and parameters optimized to provide maximal ion count for “parent” and collision-produced (“daughter”) MS/MS ions. Parent/daughter quantifier [qualifier] ions utilized: GSK205 (401.1/280[370]), 16-8 (400.1/279.1[91.1], 16-16 (387.1/280[105]), 16-18 (415.2/280[370], 16-19 (414.1/279.2[91.1]. Standard (analyte of interest)/internal standard pairs utilized: GSK205/16-16, 16-8/16-16, 16-18/GSK205, 16-19/16-8.

Calibration samples (n = 6) were prepared by adding pure standard of the measured compound to tissue homogenate (tissue + 2-fold excess water, mass/vol) in the appropriate range needed for the particular dosing regime. Organs studied were analyzed alongside the study samples. The following are typical ranges used (the lower value representing also the LLOQ at 80% accuracy limit, all other calibrator levels at 85% accuracy limit): 0.38–6 nM (plasma), 6–100 nM (skin), 6–48 nM (heart), 7.5–120 nM (brain), 19–300 or 1500–24000 nM (liver), 56–900 or 1500–12000 nM (kidney), 500–8000 nM (fat). Peak integration, calibration, and quantification was performed within Analyst software. The response of the peak area standard/int. std. to nominal concentration was linear with r = 0.999 or better.

### Patch Clamp Recordings

Heterologously transfected N2a cells were subjected to patch clamp electrophysiological recordings. Briefly, 24 h after transfection cells were prewashed with extracellular fluid (ECF) which contained (in mM) 1 MgCl_2_, 10 Glucose, 10 HEPES, 145 NaCl and 2 CaCl_2_ (pH 7.4, 310mOsM). Cells were then incubated with or without TRPV4 inhibitors in ECF for 5 min before whole cell recording. Cover slips were transferred to a recording chamber mounted on the stage of a Leica inverted microscope that was equipped with fluorescent filters. Transfected cells were identified before patching by their green florescent color. Cells were patched with a 2.5–3.0 MΩ glass electrode pulled from borosilicate glass capillaries using pipette puller (Sutter instruments). The intracellular solution contained (mM) 140 CsCl, 10 HEPES, 1 EGTA, 0.3 Na-GTP, 2 Na_2_-ATP, and 2 MgCl_2_ (pH 7.4, 295 mOsm). Whole cell currents were recorded using pclamp 9.2 software and Axopatch 200B amplifier (Molecular Devices). The cells were first clamped at −65 mV before applying a 1 s voltage ramp from −110 mV to +120 mV. The voltage ramp was applied every 2 seconds for 15 to 20 sweeps. Capacitance was monitored throughout the experimental recordings. Reported data was within ±3 pF.

### Statistical Analysis

Data are expressed as mean ± SEM. Two-tail *t*-tests or one-way ANOVA followed by Tukey *post-hoc* test were used for group comparisons. *P* < 0.05 indicated statistically significant differences

## Additional Information

**How to cite this article**: Kanju, P. *et al.* Small molecule dual-inhibitors of TRPV4 and TRPA1 for attenuation of inflammation and pain. *Sci. Rep.* **6**, 26894; doi: 10.1038/srep26894 (2016).

## Supplementary Material

Supplementary Information
