# Supplementary material for: Small molecule dual-inhibitors of TRPV4 and TRPA1 for attenuation of inflammation and pain
Source: Sci Rep. 2016 Jun 1;6:26894. doi: 10.1038/srep26894 (PMC4887995; doi:10.1038/srep26894)
Supplement: Supplementary Information [file srep26894-s1.pdf]

Supplementary File

Title:

Small molecule dual-inhibitors of TRPV4 and TRPA1 for attenuation of inflammation and pain

Authors:

Patrick Kanju[1], Yong Chen[1], Whasil Lee[1], Michele Yeo[1], Suk Hee Lee[1], Joelle Romac[2], Rafiq Shahid[2], Ping Fan[2], David M Gooden[3], Sidney A Simon[4], Ivan Spasojevic[2], Robert A. Mook Jr[2,3], Rodger A. Liddle[2], Farshid Guilak[5], Wolfgang B. Liedtke[1,4,6,7]

Affiliations:

Duke University, Durham NC USA

1 Dept of Neurology

2 Dept of Medicine

3 Dept of Chemistry

4 Dept of Neurobiology

6 Dept of Anesthesiology

7 Neurology Clinics for Headache, Head-Pain and Trigeminal Sensory Disorders

Washington University in St Louis and Shriners Hospitals for Children, St Louis MO USA

5 Department of Orthopaedic Surgery

Corresponding author WBL; correspondence to wolfgang@neuro.duke.edu

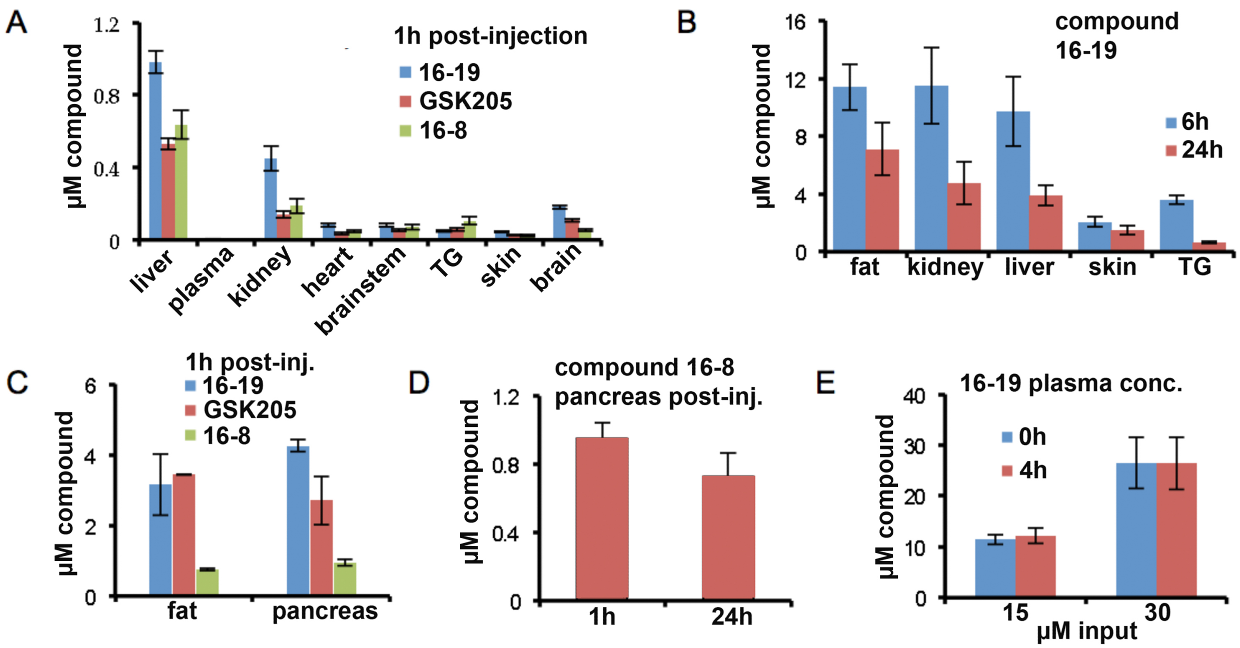

Suppl Fig. 1: *Pharmaco-kinetics/pharmaco-tox of compounds 16-8, 16-19 and GSK205 in-vivo*

(A) concentrations of compounds 16-8, 16-19 and GSK205 (10mg/kg) in several murine tissues/organs 1h post-i.p. injection. **(B)** Compound 16-19 time-course at 6h and 24h in several organs. 16-19 was selected because of its elevated levels at the 1h time-point, and based on the estimate that 16-19 is more lipophilic than 16-8 and GSK205. Note that metrics at 6h are invariably higher than at 24h. All values are appreciably higher than at 1h. **(C)** Concentrations of 16-8, 16-19 and GSK205 in fat and pancreas after one hour. Note lower concentration of 16-8 vs. 16-19 and GSK205, yet above its IC50. **(D)** Concentrations of 16-8 in the pancreas at 1h and 24h time-points. **(E)** Structural stability of compound 16-19 in plasma as suggested by stable concentration after 4h/37˚C. Results are expressed as means±SEM, n=6 mice/experimental group for all expts.

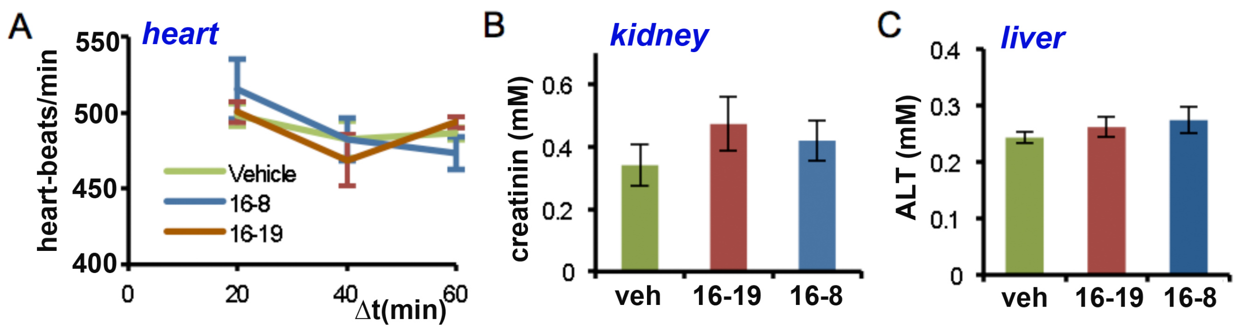

Suppl Fig. 2: *Absence of cardiac, renal and hepatic toxicty of compounds 16-8, 16-19*
(A) Heart rate time-course after i.p. injection (10mg/kg) of compounds. There was no significant difference in heart rates between vehicle and compounds. **(B)** Serum creatinin was not significantly elevated in animals treated with 16-19 and 16-8 vs vehicle control. **(C)** Serum alanin-amino-transferase (ALT) levels were not significantly elevated in animals treated with 16-19 and 16-8 vs vehicle control. n=6 mice/group for all expts.

Suppl Table 1

Properties GSK205, 16-8 and 16-19

**Compound**	**MW**	**PSA**	**c-logP(oct/H_2O)**
GSK205	400	37.53	5.35
16-8	399.6	24.4	6.33
16-19	413.6	24.2	6.58

MW – molecular weight
PSA – polar surface area
c-logP(oct/H_2O) indicates: calculated logP(octanol solubility / H_2O solubility)

Suppl Fig 3 General synthetic scheme for compounds 16-08 to 16-19.

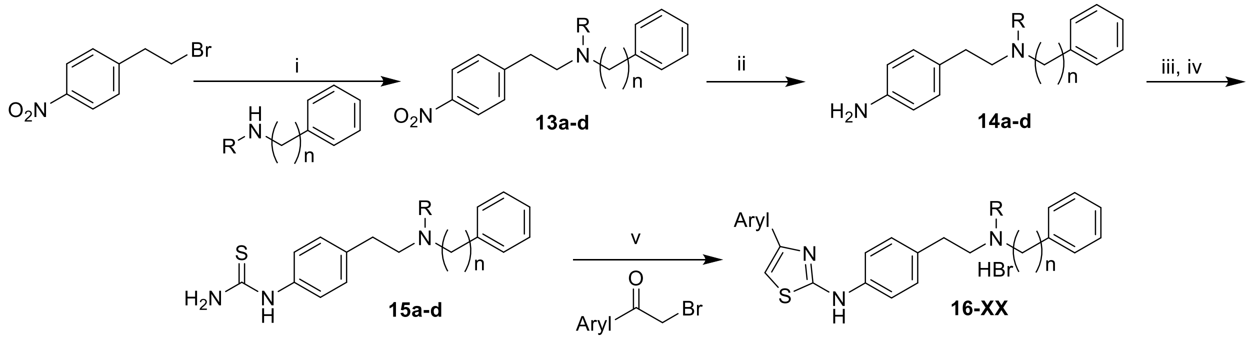

Reagents and conditions: (i) K_2CO_3, CH_3CN. (ii) Zn, MeOH, 12M HCl. (iii) 1,1′-Thiocarbonyldiimidazole. (iv) 7M NH_3 in MeOH. (v) EtOH, reflux

General procedure for the S_N2 displacement of 4-nitrophenethyl bromide: Powdered, oven-dried K_2CO_3 (1.5 eq.) and the amine (1.5 eq.) were added sequentially to a room temperature solution of the bromide (0.33 M) in anhydrous CH_3CN. The reaction mixture was heated to 80 °C (oil bath temp) until analysis of the reaction mixture by LCMS indicated complete consumption of the bromide (~6-18h). The mixture was cooled to room temperature and diluted with brine (two volume equivalents). The resulting emulsion was extracted with EtOAc (2 x one volume equivalent). The combined extracts were added to silica gel (mass of silica gel = 2x mass of starting bromide) and the mixture was concentrated to dryness under reduced pressure. Flash column chromatography (RediSepR$_f$ SiO_2, 100% CH_2Cl_2→ 5% MeOH in CH_2Cl_2) gave the product as a brown to amber oil.

Suppl Table 2. Yield of tertiary amines 13a-d

Entry	Compound number	R	n	yield
1	13a	Me	0	17%
2	13b	Me	1	49%
3	13c	Me	2	42%
4	13d	Et	1	15%

General Procedure for the nitro to aniline reduction: A solution of the nitro compound (0.5 M in MeOH) was cooled in an ice-NaCl bath. Zinc dust (4.5 eq.) was added in one portion followed by drop wise addition of 12M HCl (4.5 eq.) over 2-3 minutes. After 1h, the cooling bath was removed and the reaction mixture was allowed to stir over night at room temperature. The following morning, the mixture was cooled in an ice-NaCl bath once again and 30% aqueous NaOH was added drop wise until pH 14 (universal indicating pH paper) was reached. The mixture was diluted with CH_2Cl_2 (five volume equivalents) and stirred for 5 minutes. After this time, insolubles were removed at the vacuum and the filter cake was washed with CH_2Cl_2 (2 x 25 mL). The organic phase of the filtrate was separated, washed with brine (100 mL) and dried ($MgSO_4$). The drying agent was removed by filtration. Silica gel (~5g) was added and the filtrate was concentrated to dryness under reduced pressure. Flash column chromatography (RediSepR_f SiO_2, 100% CH_2Cl_2→ 5% MeOH in CH_2Cl_2) gave the product as a clear, amber oil.

Suppl Table 3. Yield of anilines 14a-d

Entry	Compound number	R	n	yield
1	14a	Me	0	75%
2	14b	Me	1	84%
3	14c	Me	2	97%
4	14d	Et	1	85%

General procedure for thiourea formation: A solution of the aniline (0.22 M) in anhydrous CH_2Cl_2 was added drop wise over 2-5 minutes to an ice-NaCl bath cooled solution of 1,1'-thiocarbonyldiimidazole (2 eq., 0.15 M) in anhydrous CH_2Cl_2. After 15 minutes, the cooling bath was removed and the reaction mixture was stirred at room temperature until analysis by TLC (5% MeOH in CH_2Cl_2) indicated complete consumption of the starting aniline. The mixture was cooled once again in an ice bath and 7M NH_3 in MeOH (10.5 eq.) was added drop wise over 2-5 minutes. The bath was removed and the mixture was stirred over night at room temperature. Silica gel (mass of silica gel = 2x mass of starting aniline) was added and the mixture was concentrated to dryness under reduce pressure. Flash column chromatography (RediSepR_f SiO_2, 100% CH_2Cl_2 → 10% MeOH in CH_2Cl_2) gave the pure thiourea.

Suppl Table 4. Yield of thioureas 15a-d

Entry	Compound number	R	n	yield
1	15a	Me	0	99%
2	15b	Me	1	96%
3	15c	Me	2	88%
4	15d	Et	1	67%

General procedure for thiazole formation: A mixture of the thiourea (0.1 M) in EtOH and the α-bromoacetophenone derivative (1.1 eq.) was heated to 75 oC (oil bath temperature) until analysis by TLC (5% MeOH in CH2Cl2) indicated complete consumption of the thiourea. Silica gel (mass of silica gel = 2x mass of starting thiourea) was added and the mixture was concentrated to dryness under reduce pressure. Flash column chromatography (RediSepRf SiO2, 100% CH2Cl2 → 10% MeOH in CH2Cl2) gave the pure thiazole hydrobromide**.**

Suppl Table 5. Yield of thiazole hydrobromides 16-08 to 16-19

Entry	Compound number	R	n	aryl	yield
1	16-08	Me	1	phenyl	56%
2	16-12	Me	2	3-pyridyl	82%
3	16-13	Me	1	4-pyridyl	83%
4	16-14	Me	1	2-pyridyl	94%
5	16-16	Me	0	3-pyridyl	98%
6	16-18	Et	1	3-pyridyl	31%
7	16-19	Et	1	phenyl	93%
8	16-43C	Me	1	3-pyridyl	52%
